# Environmental Screening for the *Scedosporium apiospermum* Species Complex in Public Parks in Bangkok, Thailand

**DOI:** 10.1371/journal.pone.0159869

**Published:** 2016-07-28

**Authors:** Natthanej Luplertlop, Potjaman Pumeesat, Watcharamat Muangkaew, Thanwa Wongsuk, Ana Alastruey-Izquierdo

**Affiliations:** 1 Department of Microbiology and Immunology, Faculty of Tropical Medicine, Mahidol University, Bangkok, 10400, Thailand; 2 Department of Medical Technology, Faculty of Science and Technology, Bansomdejchaopraya Rajabhat University, Bangkok, 10600, Thailand; 3 Department of Clinical Pathology, Faculty of Medicine, Vajira Hospital, Navamindradhiraj University, Bangkok, 10300, Thailand; 4 Center for Emerging and Neglected Infectious Diseases, Mahidol University, Salaya Campus, Nakorn Pathom, 73170, Thailand; 5 National Centre for Microbiology, Instituto de Salud Carlos III, Majadahonda, 228220, España; V.P.Chest Institute, INDIA

## Abstract

The *Scedosporium apiospermum* species complex, comprising filamentous fungal species *S*. *apiospermum* sensu stricto, *S*. *boydii*, *S*. *aurantiacum*, *S*. *dehoogii* and *S*. *minutispora*, are important pathogens that cause a wide variety of infections. Although some species (*S*. *boydii* and *S*. *apiospermum*) have been isolated from patients in Thailand, no environmental surveys of these fungi have been performed in Thailand or surrounding countries. In this study, we isolated and identified species of these fungi from 68 soil and 16 water samples randomly collected from 10 parks in Bangkok. After filtration and subsequent inoculation of samples on Scedo-Select III medium, colony morphological examinations and microscopic observations were performed. *Scedosporium* species were isolated from soil in 8 of the 10 parks, but were only detected in one water sample. Colony morphologies of isolates from 41 of 68 soil samples (60.29%) and 1 of 15 water samples (6.67%) were consistent with that of the *S*. *apiospermum* species complex. Each morphological type was selected for species identification based on DNA sequencing and phylogenetic analysis of the β-tubulin gene. Three species of the *S*. *apiospermum* species complex were identified: *S*. *apiospermum* (71 isolates), *S*. *aurantiacum* (6 isolates) and *S*. *dehoogii* (5 isolates). In addition, 16 sequences could not be assigned to an exact *Scedosporium* species. According to our environmental survey, the *S*. *apiospermum* species complex is widespread in soil in Bangkok, Thailand.

## Introduction

The *Scedosporium apiospermum* species complex is a group of emerging fungal pathogens that can infect both immunocompromised and immunocompetent individuals. As classified by the European Confederation of Medical Mycology/International Society for Human and Animal Mycology [1], the complex comprises five species: *S*. *apiospermum* sensu stricto, *S*. *boydii* (= *Pseudallescheria boydii*), *S*. *aurantiacum*, *S*. *dehoogii* and *S*. *minutispora*. These filamentous fungi cause a wide spectrum of infections, ranging from those that are superficial and affecting only soft tissue to invasions of tendons, ligaments, bones and internal organs as well as disseminated infections [2–4]. In addition, members of this complex have been isolated from cystic fibrosis patients [5,6]. In Thailand, *S*. *apiospermum* co-infected with *Phaeoacremonium parasiticum* has been isolated from the brains of renal transplant patients who received intravenous liposomal amphotericin B (Larbcharoensub et al., 2013).

*Scedosporium apiospermum* individuals exist as saprophytes in soil and are especially abundant in human-impacted areas, such as urban playgrounds, agricultural soils, sewage and polluted water [2,3,6,7]. Kaltseis et al. [7] investigated the occurrence of *Scedosporium* species in natural and human-dominated environments in Austria and the Netherlands. They found that the abundance of *Scedosporium* species was correlated with increasing nitrogen concentration (*P* < 0.01) and decreasing pH (*P* < 0.05) within a pH range of 6.1–7.5, whereas no significant differences were noted with respect to frequency. In addition, a positive correlation was observed between the highest concentration of ammonium and the number of *Scedosporium* strains in human-dominated environments (i.e., industrial areas, parks and playgrounds), with the latter differing from the distribution of species in clinical settings. The authors suggested that the different species have different degrees of virulence. In Australia, Harun et al. [2] undertook a qualitative environmental survey of rural and urban sites to estimate the prevalence of strains of *Scedosporium* species in the environment. They found a close association between the density of fungi recovered and degree of human activity. In a similar study in France, Rougeron et al. [6] isolated the highest densities of the *S*. *apiospermum* species complex from human-impacted areas, i.e., agricultural areas, wastewater treatment plant fluids, playgrounds and industrial areas. The *S*. *apiospermum* complex was not detected in soil samples collected from forests. Most soil samples that cultured positive for the *S*. *apiospermum* species complex exhibited pH values in the range of 6 to 8.

Because antifungal susceptibilities differ among *S*. *apiospermum* complex members [8–10], species identification is important. In addition to morphological and physiological observations, molecular techniques involving genes such as β-tubulin and calmodulin and internal transcribed spacer (ITS) regions 1 and 2 have been applied for species identification [6,10,11].

Although members of the *S*. *apiospermum* species complex have been isolated from patients in Thailand, no environmental surveys for this fungal pathogen have been carried out in Thailand or surrounding countries in Asia. In this study, we therefore investigated the environmental distribution of the *S*. *apiospermum* species complex in Bangkok, Thailand, by collecting soil and water samples from 10 city parks. For species identification, we performed DNA barcoding with reference strains. Our study is the first reported environmental survey of the *S*. *apiospermum* species complex in Thailand.

## Materials and Methods

### Ethic Statement

All locations were received formal authorization and granted permission for collecting samples from each park by Director of Division of Environment, Bangkok, Thailand.

### Soil Sampling

Sixty-eight soil samples were randomly collected from 10 parks in Bangkok (Table 1). Three to ten 1-m^2^ sites were sampled in each park, with soil samples obtained from four positions per site. Soil was collected from a depth of approximately 15 cm using a sterile metal spoon to avoid plant debris, weeds or branches. Samples were placed in sterile plastic bags and stored at 4°C until processed.

**Table 1:** Sample collection areas in Bangkok, Thailand.

Park	District	Geographical coordinates (Latitude-Longitude)	No. of sampled sites (soil/water)	No. of positive cultures (soil/water)	No. of sequenced isolates (soil/water)	Distribution of *Scedosporium apiospermum* species complex members			
						*S*. *apiospermum*	*S*. *aurantiacum*	*S*. *dehoogii*	Unidentified species
Chatuchak	Chatuchak	13.80N-100.55E	10/1	5/1	19/1	13	3	1	3
Wachirabenchatat	Chatuchak	13.81N-100.55E	10/1	8/0	19/0	12	0	0	7
Queen Sirikit	Chatuchak	13.80N-100.55E	10/1	7/0	16/0	13	0	0	3
Santiphap	Ratchathewi	13.76N-100.54E	3/0	2/0	8/0	6	0	2	0
Sirintrapruksapan	Bangkok Noi	13.74N-100.46E	5/2	0/0	0/0	0	0	0	0
His Majesty the King’s 80th Birthday Anniversary	Bangkok Noi	13.77N-100.46E	5/1	0/0	0/0	0	0	0	0
Suan Luang Rama IX	Prawet	13.68N-100.66E	10/3	6/0	14/0	13	0	0	1
Phra Nakhon	Lat Krabang	13.71N-100.78E	5/2	4/0	10/0	6	1	1	2
Nong Chok	Nong Chok	13.85N-100.85E	5/1	4/0	4/0	4	0	0	0
Thonburirom	Tungkru	13.65N-100.49E	5/3	5/0	7	4	2	1	0

### Isolation of the *S*. *apiospermum* Species Complex from Soil

Fungal isolation was performed according to Rougeron et al. [6] with slight modifications. Soil samples (5 g) were suspended in 15 ml sterile distilled water, mixed vigorously and filtered through 100-μm nylon cell strainers (Falcon, Durham, NC, USA). The filtrate was centrifuged at 7,000 ×*g* for 5 min. After discarding supernatants, pellets were re-suspended in 5 ml distilled water. Finally, 100-μl aliquots of suspension from each sample were inoculated onto five plates of Scedo-Select III medium. This medium, which was chosen because it prevents or minimizes the growth of other rapidly growing fungal species, was prepared according to Pham et al. [12]. Plates were incubated at 35°C for 5 days and colony morphology was investigated.

### Sampling and Isolation of the *S*. *apiospermum* Species Complex from Water

Fifteen pond water samples (500 ml) were randomly collected in sterile bottles from the 10 parks in Bangkok. pH measurements were taken using pH test paper (Toyo Roshi, Tokyo, Japan). One hundred milliliters of each water sample was filtered through white gridded 0.45-μm, 47-mm S-Pack membrane filters (Merck Millipore, Darmstadt, Germany) and placed on five plates of Scedo-Select III medium. After incubating the plates at 35°C for 5 days, colony morphology was investigated.

### Measurements of Physicochemical Parameters

Soil pH and ammonium, nitrate, potassium and phosphorus concentrations were measured using a soil test kit for N, P, K and pH (Ecoagro, Bangkok, Thailand).

### Morphological Identification of the *S*. *apiospermum* Species Complex

Colony morphologies were observed visually and microscopically according to Gilgado et al. [10,13] and compared with the following standard strains obtained from el Servicio de Micología, Instituto de Salud Carlos III, Madrid, Spain: *S*. *dehoogii* CM 4798, *S*. *angusta* CBS 254.72 (= *Pseudallescheria angusta*), *S*. *apiospermum* CBS 117410, *S*. *boydii* CBS 120157 and *S*. *aurantiacum* CBS 116910. We hypothesized that soil and water samples could contain multiple strains of the *S*. *apiospermum* species complex, which might manifest as different colony morphologies on solid agar. In view of this possibility, a single colony of each morphological type on a given plate was selected for further analysis.

Colonies identified as the *S*. *apiospermum* species complex were inoculated on Scedo-Select III for purification. The colonies isolated from Scedo-Select III were then collected and inoculated onto Sabouraud dextrose agar for macroscopic and microscopic observation.

### Genus and Species Identification of the *S*. *apiospermum* Species Complex by DNA Sequencing and Analysis

Standard strains and strains isolated from soil and water were inoculated into peptone dextrose broth and incubated at 35°C for 7 days. DNA was extracted with an EZNA Fungal DNA mini kit (Omega Bio-tek) and PCR-amplified with β-tubulin gene-specific primers (TUB-F: 5′-CTGTCCAACCCCTCTTACGGCGACCTGAAC-3′; TUB-R: 5′ ACCCTCACCAGTATACCAATGCAAGAAAGC-3′) [6,14]. Each 50-μl reaction mixture contained 2× GoTaq Colorless Master Mix (Promega, USA), 0.5 μM of each primer, nuclease-free water and DNA template. PCR amplifications were carried out in a T100 Thermal Cycler (Bio-Rad) according to the following protocol: preheating at 96°C for 6 min, followed by 35 cycles at 94°C for 1 min, 56°C for 1 min, and 72°C for 45 s, and a final extension step at 72°C for 10 min. Five microliters of the resulting PCR products were electrophoretically separated on a 1.5% agarose gel in 1× TBE buffer, stained with 1 μg ml^−1^ ethidium bromide, and photographed using a Gel Doc XR+ system (Bio-Rad).

PCR products were purified and bidirectionally sequenced by Macrogen (Seoul, South Korea). High-quality sequences were obtained from 98 environmental isolates and 5 isolates of control strains. The retrieved sequence files were edited and subjected to pairwise alignment using BioEdit software (http://www.mbio.ncsu.edu/bioedit/bioedit.html). Edited sequences were compared with existing sequences in GenBank using BLASTn (http://blast.ncbi.nlm.nih.gov/Blast.cgi). The generated nucleotide sequences were deposited in GenBank under accession numbers KU53533641 to KU533723 and KX382894 to KX382909

### Phylogenetic Analysis

The bioinformatics data were analyzed and used to assess inter- and intra-species nucleotide variation of the β-tubulin gene in all strains analyzed in this study. To assess evolutionary relationships among isolates, all 103 generated β-tubulin gene sequences as well as 13 sequences of *S*. *apiospermum* and related species downloaded from GenBank were multiply aligned using BioEdit. The downloaded sequences were as follows: KC812577.1, KC812581.1, KC779459.1, JQ691052.1, KC779501.1, JQ691042.1 and KT186641.1 (*S*. *apiospermum*), KC812551.1 (*S*. *boydii*), AJ890124.1 (*Pseudallescheria ellipsoidea*), AJ890131.1 (*Pseudallescheria fusoidea*), JQ691062.1 (*S*. *minutispora*), AJ890127.1 (*Lomentospora prolificans* [= *Scedosporium prolificans*]) and AJ890132.1 (*Petriellopsis africana* [= *Pseudallescheria africana*]). A phylogenetic tree of the 116 aligned sequences excluding gaps and missing data was constructed by maximum likelihood based on the Tamura–Nei model in MEGA6 (Tamura et al., 2013). Initial tree(s) for the heuristic search were obtained by applying the neighbor-joining method to a matrix of pairwise distances estimated using the maximum composite likelihood approach. A bootstrap analysis was conducted with 1,000 replications.

## Results

### Characterization of the Culturable *S*. *apiospermum* Species Complex Population in Soil

To aid our morphological examinations, we observed the colony morphology of standard strains, i.e., *S*. *dehoogii* CM 4798, *S*. *angusta* CBS 254.72, *S*. *apiospermum* CBS 117410, *S*. *boydii* CBS 120157 and *S*. *aurantiacum* CBS 116910, on days 1, 3, 5, 7, 9 and 11 of incubation at 35°C on Scedo-Select III agar (S1 File).

**Fig 1:**
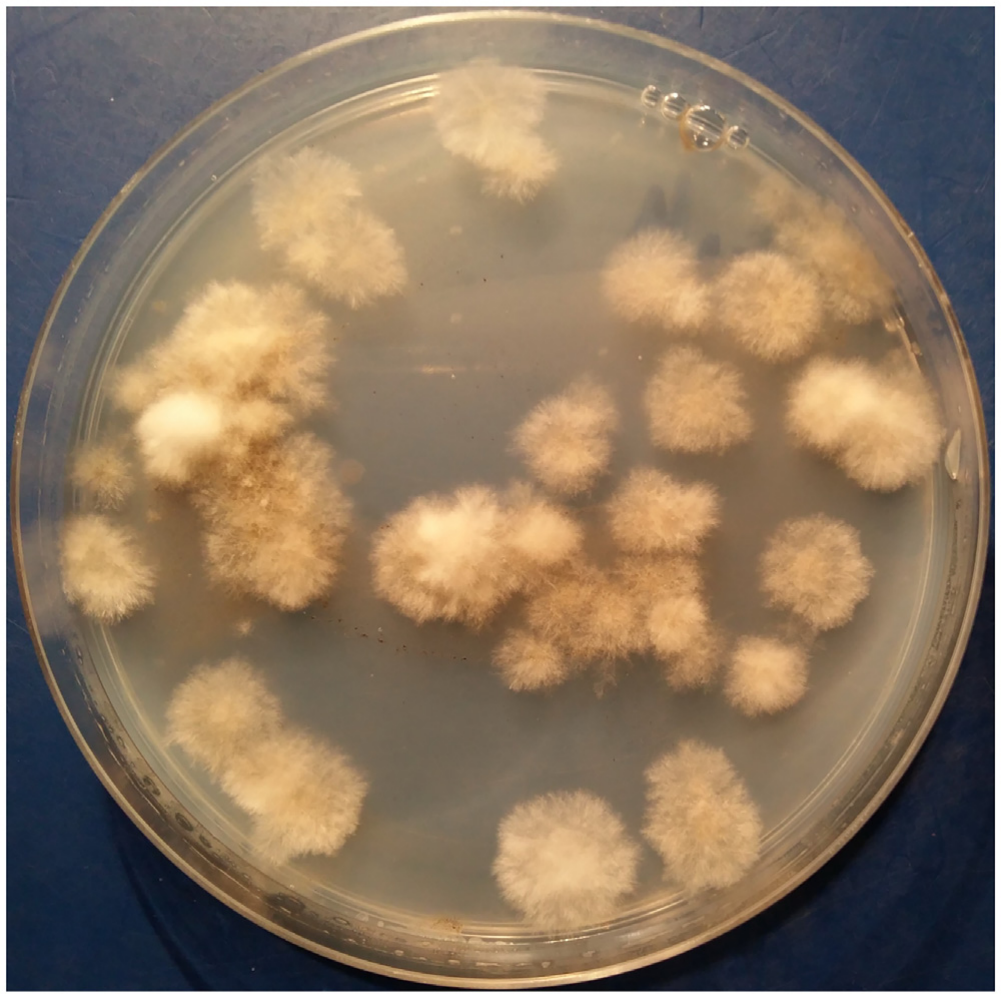
Colonies of *Scedosporium* on Scedo-Select III agar after inoculation of 100 μl of soil suspension from each sample and incubation at 35°C for 5 days.

As shown in Table 1, the *S*. *apiospermum* species complex could be isolated from soil collected from eight of the 10 sampled parks. Only one water sample, with a pH of 6.0, yielded fungus identifiable as *Scedosporium*. The pH of the soil samples ranged between 6.0 and 7.5, with ammonium, nitrate, phosphorus and potassium concentrations varying from 0–10, 0–30, 1–12 and 0–40 mg kg^−1^, respectively. As shown in Fig 1, we found numerous colonies with different morphologies on each Scedo-Select III agar plate. From each plate, we selected one representative colony of each morphological type of *Scedosporium*, which yielded 98 single colonies: 97 colonies from 41 of 68 soil samples and only 1 colony from 1 of 15 water samples. These *Scedosporium* colony isolates, which were subsequently evaluated by PCR amplification and sequencing of the β-tubulin gene, are listed in S1 Table.

### Molecular Identification Based on the β-Tubulin Gene

PCR amplification of the β-tubulin gene was successful for all strains, with a single band of approximately 650 bp observed on gels following electrophoresis. Each generated β-tubulin nucleotide sequence was searched against sequences in the NCBI database using the BLASTn algorithm. Outputs from the BLAST searches were sorted on the basis of maximum identity. Sequence-based identities with a cutoff of 97% or greater were considered significant in this study, with the best hit defined as the sequence with the highest maximum identity to the query sequence. Three species of the *S*. *apiospermum* species complex were identified: *S*. *apiospermum* (71 isolates), *S*. *aurantiacum* (6 isolates) and *S*. *dehoogii* (5 isolates). In addition, there were 16 β-tubulin sequences that could not be assigned to a specific *Scedosporium* species.

Full-length sequences of the β-tubulin gene were obtained from all identified isolates with six exceptions: *S*. *apiospermum* isolate H52H2I2 (565 bp), *S*. *apiospermum* isolate I42H2I2 (618 bp), *S*. *apiospermum* isolate R12R1B2 (610 bp), *S*. *aurantiacum* isolate R52R1E7 (613 bp), *S*. *aurantiacum* isolate A032 (591 bp) and *S*. *dehoogii* isolate H25H1E5 (641 bp). Intra-specific length variation was observed among the remaining 76 identified sequences, although sequences were 98%–99% similar within species. In particular, the lengths of the 68 sequences of *S*. *apiospermum* were variously 653 bp (6 sequences), 654 bp (23 sequences) and 655 bp (39 sequences); the lengths of the four sequences of *S*. *aurantiacum* were 672 bp (3 sequences) and 676 bp (1 sequence), and those of the four sequences of *S*. *dehoogii* were 649 bp (1 sequence) and 654 bp (3 sequences). Because sequence lengths overlapped between *S*. *apiospermum* and *S*. *dehoogii*, gel electrophoresis could not be used for species differentiation.

**Fig 2:**
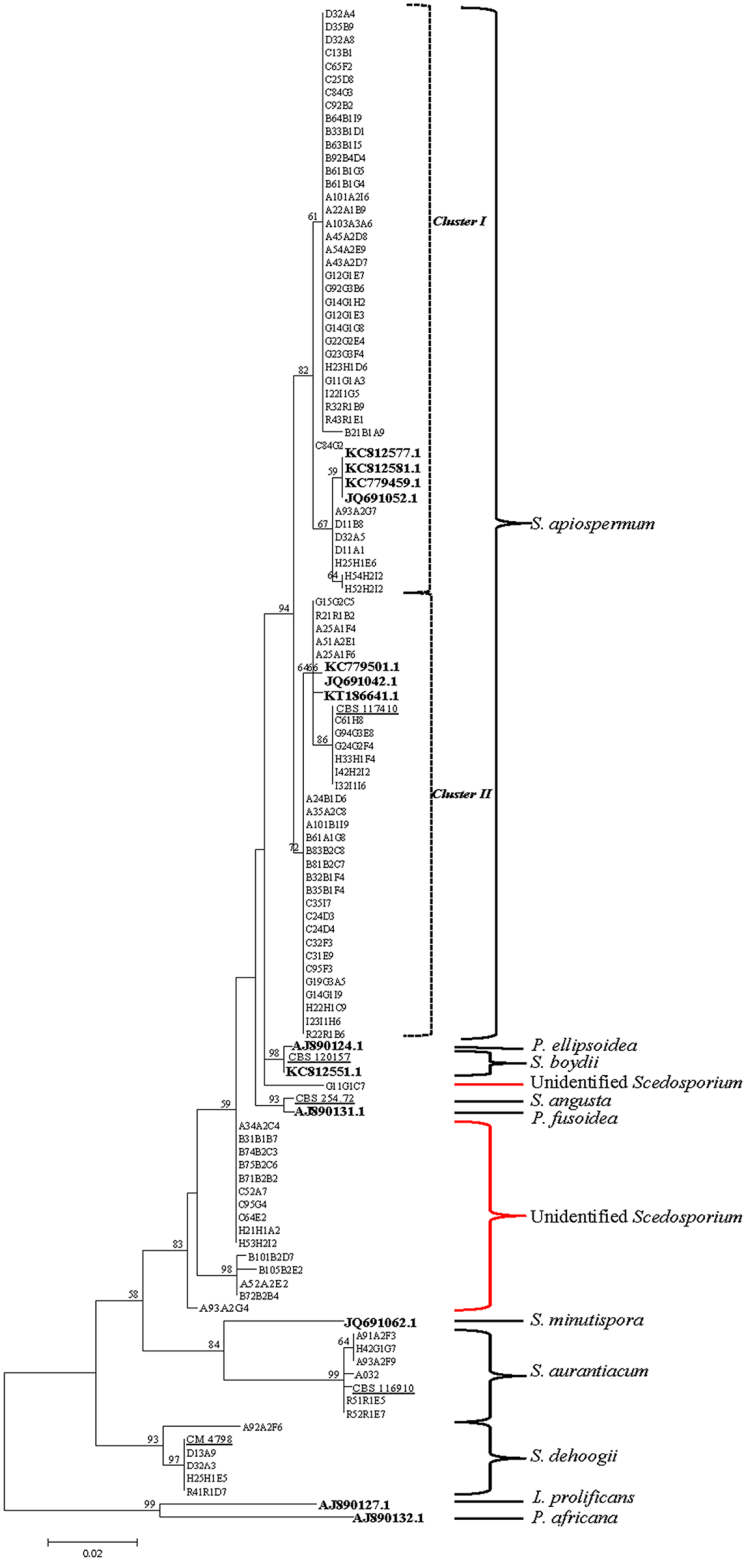
Maximum-likelihood tree of β-tubulin gene sequences of *Scedosporium* isolates and reference strains. The tree recovered under the Tamura–Nei model with the highest log likelihood (−1,599.6719) is shown. The tree is drawn to scale, with branch lengths corresponding to the number of substitutions per site. Bootstrap values ≥ 50% (1,000 replicates) are shown above branches. Accession numbers of *Scedosporium* sequences retrieved from GenBank are shown in bold; accession numbers of standard strains are underlined. *Lomentospora prolificans* and *Pseudallescheria africana* were used as outgroups. Genus abbreviations are as follows: *L*, *Lomentospora*; *P*, *Pseudallescheria*, *S*, *Scedosporium*.

Multiple alignment of all 98 β-tubulin sequences revealed significant divergences, including both substitutions and insertion/deletions, between them. Excluding gaps, there were 654 nucleotide positions in the alignment. We next aligned the 98 generated sequences with 13 β-tubulin sequences of *S*. *apiospermum* and related species downloaded from GenBank. After elimination of gaps and missing data, the final dataset of 116 sequences comprised 463 nucleotide positions. Phylogenetic analysis of this 116-sequence dataset generated the tree shown in Fig 2. In this tree, *S*. *dehoogii* (bootstrap value [BV] = 93%) was sister to a weakly supported (BV = 58%) clade comprising all other non-outgroup taxa. This latter clade was in turn divided into two moderately supported subclades. The first of these subclades (BV = 84%) consisted of *S*. *aurantiacum* and *S*. *minutispora*; the second subclade (BV = 83%) included *S*. *apiospermum*, *P*. *ellipsoidea*, *P*. *fusoidea*, *S*. *angusta* and all unidentified *Scedosporium* isolates. The isolates identified as *S*. *apiospermum* were strongly clustered together (BV = 94%) and were subdivided into two groups.

## Discussion

The natural niches of the *Scedosporium apiospermum* species complex in Thailand are currently unknown. The purpose of our study was to investigate the distribution of environmental species of the *S*. *apiospermum* species complex in Thailand by collecting soil and water from human-dominated environments, namely, 10 public parks in Bangkok. Using Scedo-Select III agar developed by Pham et al. [12], we detected *Scedosporium* species from samples having a pH range of 6.0 to 7.5. Soil samples from eight parks (i.e., Chatuchak, Wachirabenchatat, Queen Sirikit, Santiphap, Suan Luang Rama IX, Phra Nakhon, Nong Chok and Thonburirom parks) were positive for *Scedosporium* colonies. Unfortunately, we were only able to isolate one colony from water samples because there were many other fungi that grew on the inside of the membrane filter surfaces. Nevertheless, we can conclude that S*cedosporium* species are ubiquitous in soil in Bangkok, Thailand.

Notably, a substantial number of *Scedosporium* strains that were identified morphologically were confirmed by sequencing of the β-tubulin gene in this study. The only *Scedosporium* sequences from Thailand previously deposited in GenBank are six ITS sequences of *S*. *boydii* (JN116614.1, JQ409649.1, JQ409648.1, JQ409647.1, JQ409646.1 and JQ409645.1). In regard to case reports, *S*. *apiospermum* has been reported in brain abscesses of near-drowning and renal transplant patients ([15] [16]; *S*. *boydii* has been reported also [17]. Our study is thus the first to report the presence of *S*. *aurantiacum* and *S*. *dehoogii* isolates and sequences from Thailand. These latter two species have also been detected from soil in France, Austria, the Netherlands and Australia (Harun et al., 2010; Kaltseis et al., 2009; [6]. In addition, *Lomentospora prolificans* (= *Scedosporium prolificans*) has been reported in a case of disseminated infection of an acute myeloid patient with prolonged febrile neutropenia in Thailand [18]. Unfortunately, the colony of *L*. *prolificans* has not been detected in our study.

Of the 98 isolates examined in the present study, the most abundant species was *S*. *apiospermum* (73%). This finding is in agreement with the previous study of Rougeron et al. [6], who used Scedo-Select III selective culture agar and reported that *S*. *apiospermum* and *S*. *dehoogii* were the most frequently isolated species from city parks in France. Moreover, Kaltseis et al. [7] used ScedoSel+ selective culture agar and reported that *S*. *apiospermum* was the most frequently isolated species from parks and playground in Austria and the Netherlands. In contrast, *S*. *aurantiacum* was the species most frequently isolated in inner suburbs of Sydney, Australia, by Harun et al. [2], who used dichloran rose bengal chloramphenicol medium supplemented with benomyl. These discrepancies in the distribution patterns of *Scedosporium* species could be related to differences in climate or soil enrichment and/or depend on the choice of selective medium.

Phylogenetic analysis of the unidentified *Scedosporium* strains based on β-tubulin sequences placed these isolates in a clade containing *S*. *apiospermum*, *S*. *boydii*, *P*. *ellipsoidea* and *P*. *fusoidea*, with their sequences showing the greatest similarity (95%–98%) to *S*. *boydii* and *S*. *apiospermum*. We plan to conduct additional studies to refine the identity of these undetermined *Scedosporium* species.

Multiple alignment of the 654-bp β-tubulin fragment of the 98 *Scedosporium* isolates revealed 21 unique sequences among the three identified species and indicated the presence of substantial diversity within species of *Scedosporium*. An alignment of these unique sequences with sequences of five control strains and 16 unidentified *Scedosporium* isolates is shown in S2 File. Because the exon 5/6 region of the β-tubulin gene used in this study was of limited utility for resolving the relationships of the 16 unidentified *Scedosporium* strains to other analyzed species, additional DNA barcoding genes will be selected to differentiate them. According to Chen et al.[19], the standard barcoding genetic locus, ITS, can discriminate most *Scedosporium* species except for the *S*. *apiospermum* species complex, which was defined in their study as comprising *S*. *apiospermum*, *S*. *boydii* and *S*. *angusta*. Their investigation of molecular markers indicated, however, that β-tubulin was the best barcoding marker at the lineage level, with the performance of markers to resolve *Scedosporium* taxa ranked from best to worst as β-tubulin (BT2) > γ-actin > factor 1α (TEF1) > ITS (i.e., internal transcribed spacer of the small ribosomal protein 60sS L10 (L1) [19]. In future work, we will continue to search for additional markers to resolve the unidentified *Scedosporium* and add to the knowledge of *Scedosporium* taxonomy.

In summary, the *S*. *apiospermum* species complex is widespread in soil in Bangkok, Thailand. This knowledge should prove useful as we explore the possible connection between environmental sources and clinical infection in Thailand.

## Supporting Information

S1 File Colony morphologies of standard strains on Scedo-Select III. Colony morphologies of *Scedosporium dehoogii* CM 4798, *S*. *angusta* CBS 254.72, *S*. *apiospermum* CBS 117410, *S*. *boydii* CBS 120157 and *S*. *aurantiacum* CBS 116910 reference strains on days 1, 3, 5, 7, 9 and 11 after incubation at 35°C on Scedo-Select III agar.
(DOCX)

S2 File Multiple alignment of β-tubulin gene sequences. Multiple alignment of β-tubulin gene sequences of 21 unique sequences of known species of *Scedosporium*, 5 control strains and 16 unidentified *Scedosporium*. Quotation marks indicate nucleotides that are identical relative to the top-most sequence; a dash indicates an insertion/deletion situation.
(DOCX)

S1 Table *Scedosporium* isolates, which were evaluated by PCR amplification and sequencing of the β-tubulin gene. *Scedosporium* isolates subjected to β-tubulin gene sequencing. (All listed isolates were obtained from soil except for A032, which was isolated from water.)
(DOCX)
