# Supplementary material for: Environmental Screening for the Scedosporium apiospermum Species Complex in Public Parks in Bangkok, Thailand
Source: PLoS One. 2016 Jul 28;11(7):e0159869. doi: 10.1371/journal.pone.0159869 (PMC4965192; doi:10.1371/journal.pone.0159869)
Supplement: S1 File — Colony morphologies of Scedosporium dehoogii CM 4798, S. angusta CBS 254.72, S. apiospermum CBS 117410, S. boydii CBS 120157 and S. aurantiacum CBS 116910 reference strains on days 1, 3, 5, 7, 9 and 11 after incubation at 35°C on Scedo-Select III agar. (DOCX) [file pone.0159869.s001.docx]

**S1 File. Colony morphologies of standard strains on Scedo-Select III**

| Day | *S. angusta*  CBS 254.72 | *S. apiospermum*  CBS 117410 | *S. aurantiacum*  CBS 116910 | *S. boydii*  CBS 120157 | *S. dehoogii*  CM 4798 |
| --- | --- | --- | --- | --- | --- |
| Day 1 | 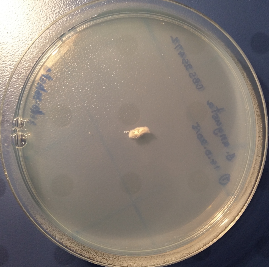 | 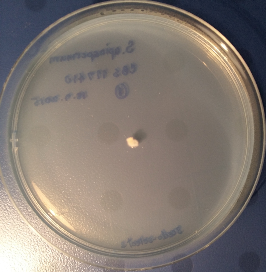 | 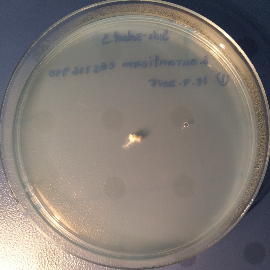 | 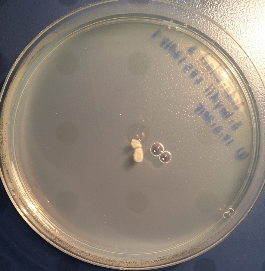 | 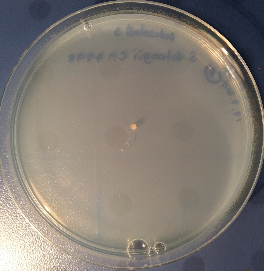 |
| Day 3 | 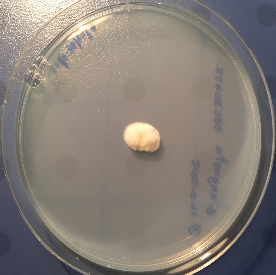 | 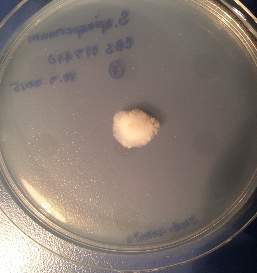 | 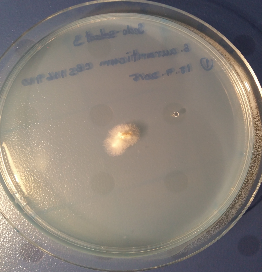 | 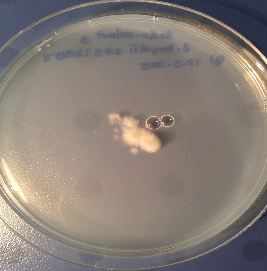 | 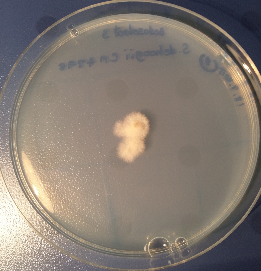 |
| Day 5 | 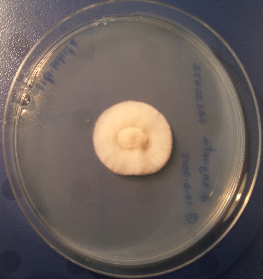 | 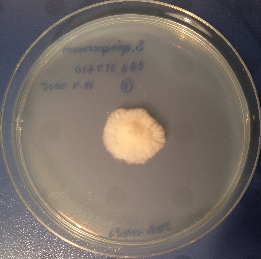 | 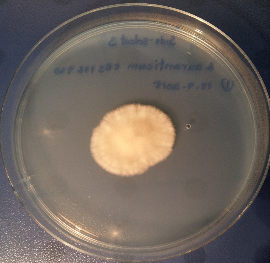 | 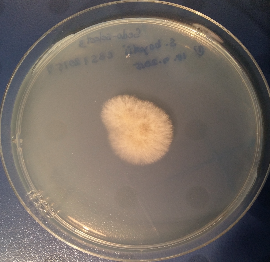 | 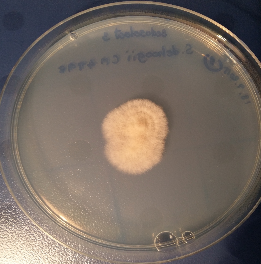 |
| Day 7 | 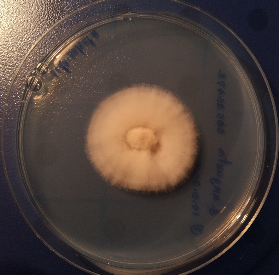 | 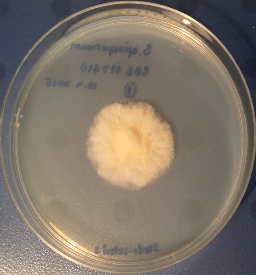 | 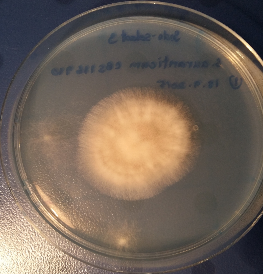 | 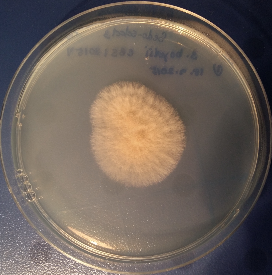 | 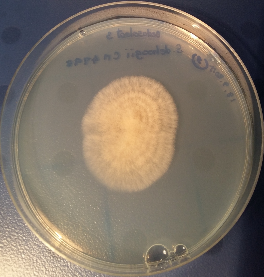 |
| Day 9 | 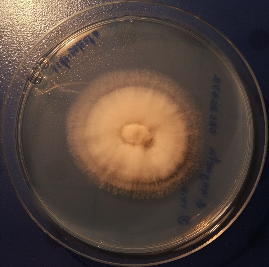 | 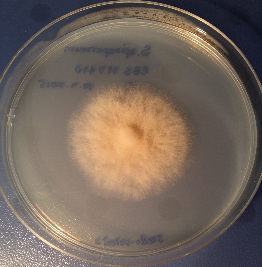 | 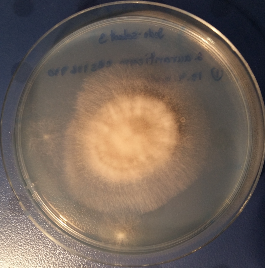 | 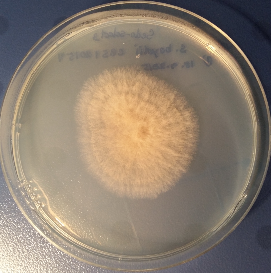 | 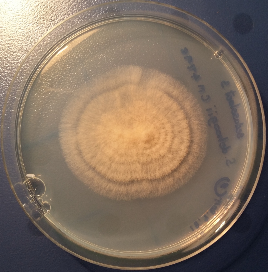 |
| Day 11 | 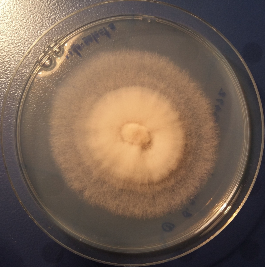 | 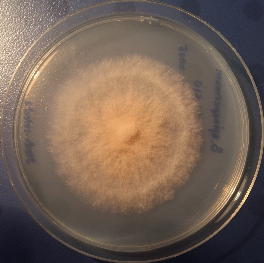 | 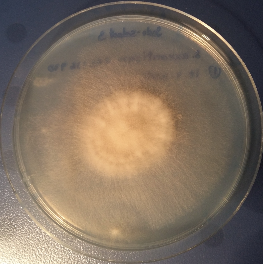 | 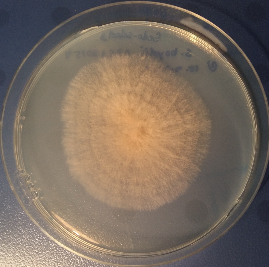 | 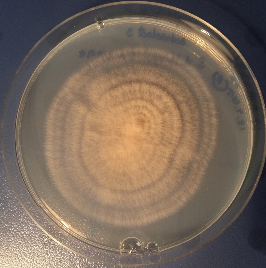 |
