# Supplementary figures and images for: Environmental Screening for the Scedosporium apiospermum Species Complex in Public Parks in Bangkok, Thailand
Source: PLoS One. 2016 Jul 28;11(7):e0159869. doi: 10.1371/journal.pone.0159869 (PMC4965192; doi:10.1371/journal.pone.0159869)

**S2 File. Multiple alignment of β-tubulin gene sequences.**


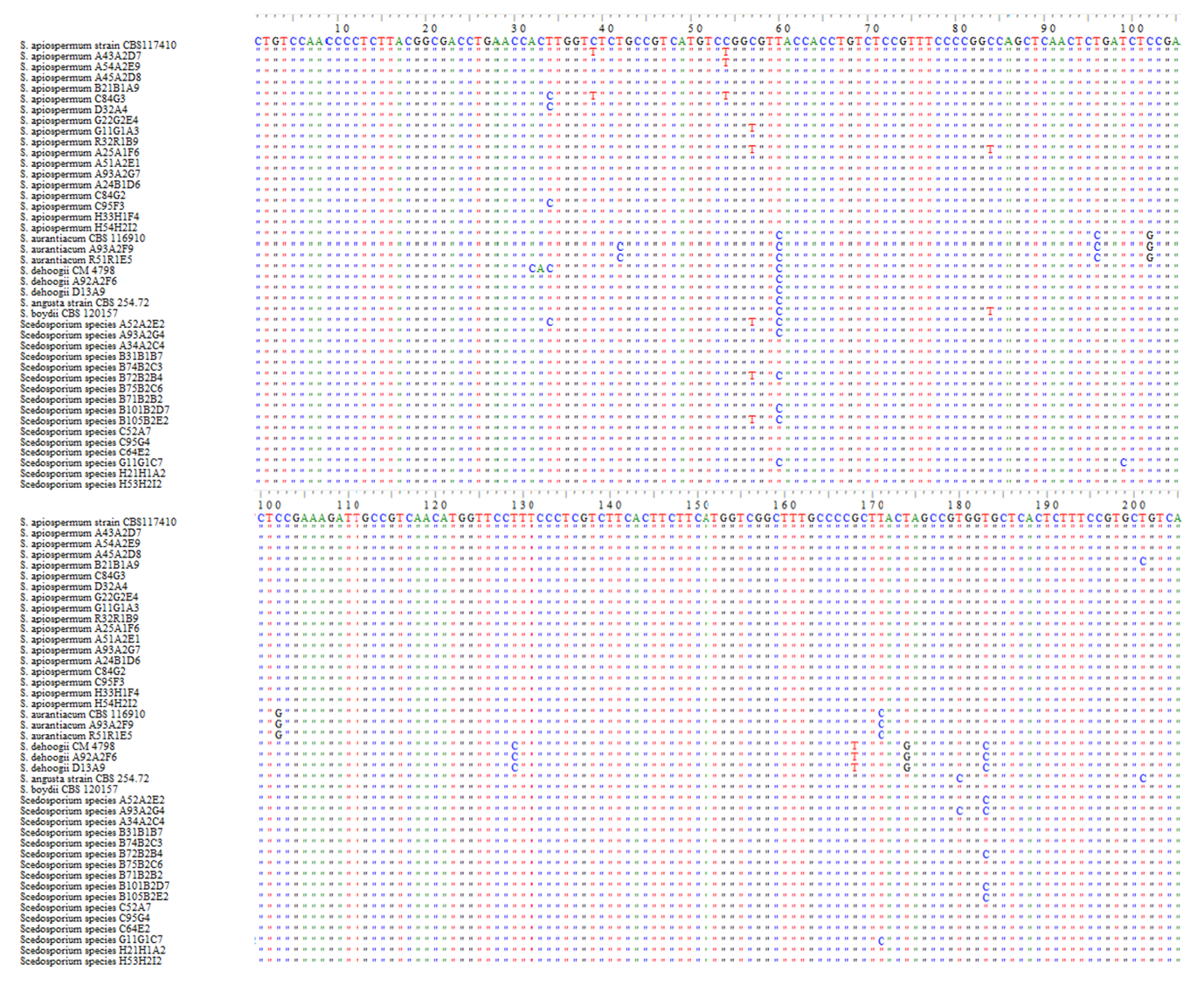


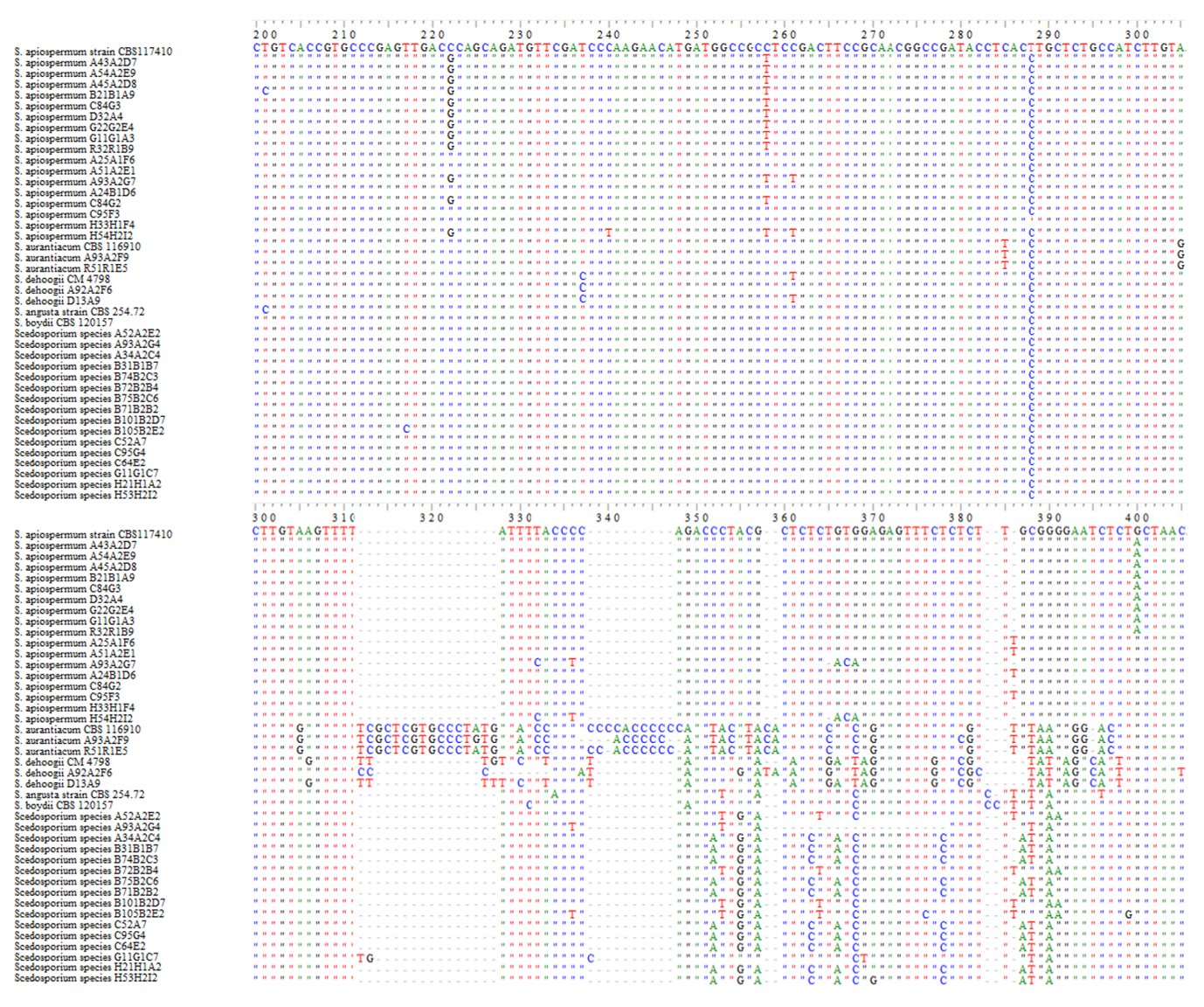


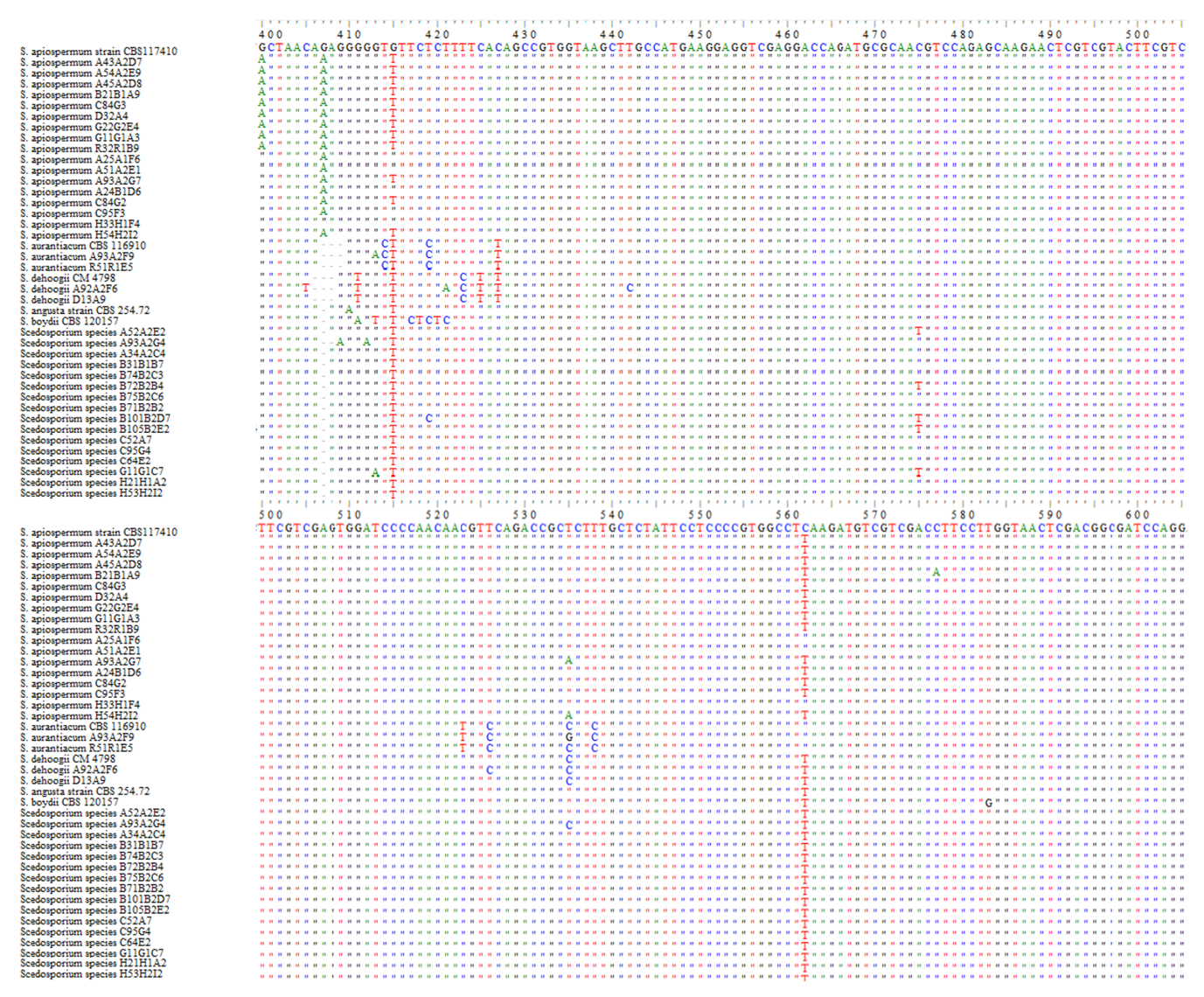


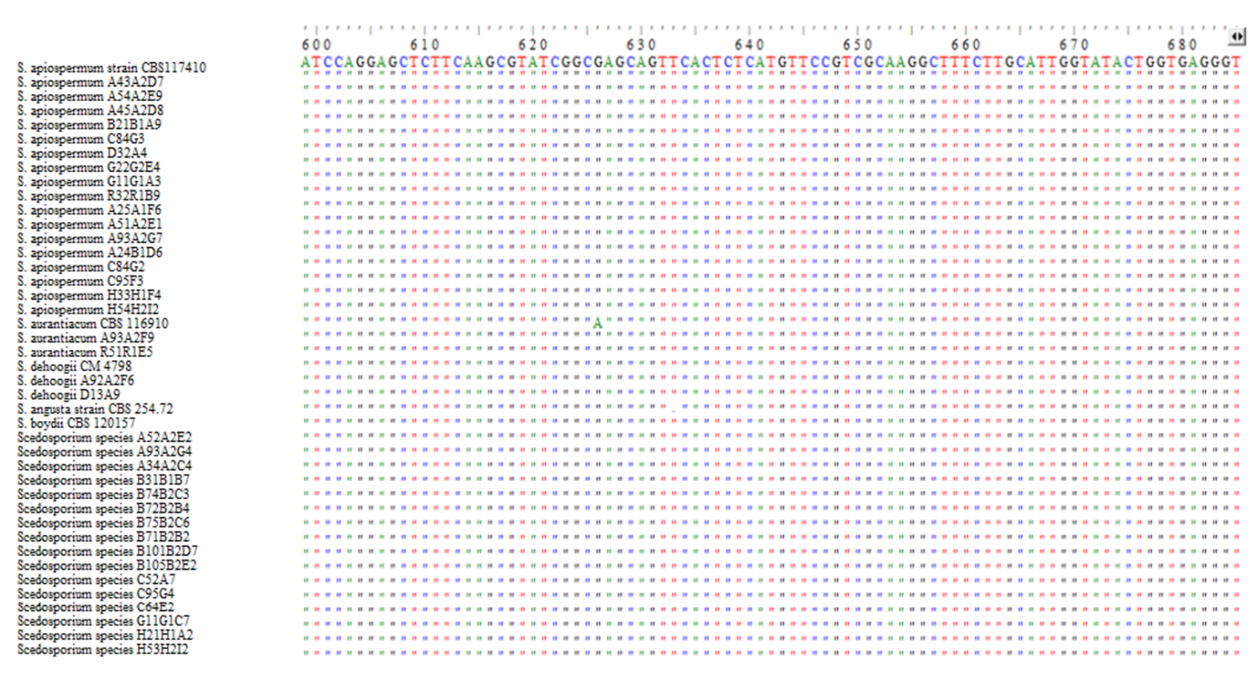

Supplement: S2 File — Multiple alignment of β-tubulin gene sequences of 21 unique sequences of known species of Scedosporium, 5 control strains and 16 unidentified Scedosporium. Quotation marks indicate nucleotides that are identical relative to the top-most sequence; a dash indicates an insertion/deletion situation. (DOCX) [file pone.0159869.s002.docx]
