# Supplementary material for: Environmental Screening for the Scedosporium apiospermum Species Complex in Public Parks in Bangkok, Thailand
Source: PLoS One. 2016 Jul 28;11(7):e0159869. doi: 10.1371/journal.pone.0159869 (PMC4965192; doi:10.1371/journal.pone.0159869)
Supplement: S1 Table — Scedosporium isolates subjected to β-tubulin gene sequencing. (All listed isolates were obtained from soil except for A032, which was isolated from water.) (DOCX) [file pone.0159869.s003.docx]

**S1 Table.** *Scedosporium* isolates subjected to β-tubulin gene sequencing. (All listed isolates were obtained from soil except for A032, which was isolated from water.)

| **Taxon inferred from sequencing results** | **Isolate** | **Latitude** | **Longitude** | **GenBank**  **Number** |
| --- | --- | --- | --- | --- |
| *S. apiospermum*  *S. apiospermum*  *S. apiospermum*  *S. apiospermum*  *S. apiospermum*  *S. apiospermum*  *S. dehoogii*  Unidentified *Scedosporium*  *S. apiospermum*  *S. apiospermum*  *S. apiospermum*  Unidentified *Scedosporium*  *S. aurantiacum*  *S. aurantiacum*  *S. apiospermum*  *S. apiospermum*  *S. apiospermum*  Unidentified *Scedosporium*  *S. apiospermum*  *S. aurantiacum*  *S. apiospermum*  Unidentified *Scedisporium*  Unidentified *Scedosporium*  *S. apiospermum*  Unidentified *Scedosporium*  *S. apiospermum*  Unidentified *Scedosporium*  Unidentified *Scedosporium*  *S. apiospermum*  *S. apiospermum*  *S. apiospermum*  Unidentified *Scedosporium*  *S. apiospermum*  *S. apiospermum*  Unidentified *Scedosporium*  *S. apiospermum*  *S. apiospermum*  *S. apiospermum*  *S. apiospermum*  *S. apiospermum*  *S. apiospermum*  *S. apiospermum*  *S. apiospermum*  Unidentified *Scedosporium*  *S. apiospermum*  *S. apiospermum*  *S. apiospermum*  Unidentified *Scedosporium*  *S. apiospermum*  *S. apiospermum*  *S. apiospermum*  *S. apiospermum*  *S. apiospermum*  *S. apiospermum*  Unidentified *Scedosporium*  *S. apiospermum*  *S. apiospermum*  *S. dehoogii*  *S. apiospermum*  *S. apiospermum*  *S. apiospermum*  *S. apiospermum*  *S. dehoogii*  *S. apiospermum*  *S. apiospermum*  *S. apiospermum*  *S. apiospermum*  *S. apiospermum*  S. apiospermum  Unidentified *Scedosporium*  *S. apiospermum*  *S. apiospermum*  *S. apiospermum*  *S. apiospermum*  *S. apiospermum*  *S. apiospermum*  *S. apiospermum*  *S. apiospermum*  *S. apiospermum*  *S. apiospermum*  *S. apiospermum*  Unidentified *Scedosporium*  *S. dehoogii*  Unidentified *Scedosporium*  *S. apiospermum*  *S. apiospermum*  *S. aurantiacum*  *S. apiospermum*  *S. apiospermum*  *S. apiospermum*  *S. apiospermum*  *S. aurantiacum*  *S. aurantiacum*  *S. apiospermum*  *S. apiospermum*  *S. apiospermum*  *S. dehoogii*  *S. apiospermum* | A43A2D7  A54A2E9  A45A2D8  A103A3A6  A25A1F6  A51A2E1  A92A2F6  A52A2E2  A25A1F4  A93A2G7  A24B1D6  A93A2G4  A93A2F9  A91A2F3  A22A1B9  A35A2C8  A101B1I9  A34A2C4  A101A2I6  A032  B61A1G8  B31B1B7  B74B2C3  B61B1G4  B72B2B4  B21B1A9  B75B2C6  B71B2B2  B61B1G5  B92B4D4  B63B1I5  B101B2D7  B33B1D1  B83B2C8  B105B2E2  B64B1I9  B81B2C7  B32B1C7  B35B1F4  C92B2  C35I7  C24D3  C24D4  C52A7  C32F3  C84G3  C61H8  C95G4  C31E9  C25D8  C65F2  C84G2  C13B1  C95F3  C64E2  D11B8  D32A8  D13A9  D32A5  D11A1  D32A4  D35B9  D32A3  G15G2C5  G19G3A5  G12G1E7  G92G3B6  G14G1H2  G12G1E3  G11G1C7  G14G1G8  G14G1I9  G94G3E8  G22G2E4  G23G2F4  G24G2F4  G11G1A3  H33H1F4  H25H1E6  H23H1D6  H22H1C9  H21H1A2  H25H1E5  H53H2I2  H54H2I2  H52H2I2  H42H1G7  I42H2I2  I23I1H6  I22I1G5  I32I1I6  R51R1E5  R52R1E7  R32R1B9  R21R1B2  R22R1B6  R41R1D7  R43R1E1 | N 13.80508°  N 13.80446°  N 13.80508°  N 13.80435°  N 13.80704°  N 13.80446°  N 13.80384°  N 13.80446°  N 13.80704°  N 13.80384°  N 13.80704°  N 13.80384°  N 13.80384°  N 13.80384°  N 13.80704°  N 13.80551°  N 13.80435°  N 13.80551°  N 13.80435°  N 13.80729°  N 13.81511°  N 13.81330°  N 13.81550°  N 13.81511°  N 13.81550°  N 13.81511°  N 13.81550°  N 13.81550°  N 13.81511°  N 13.81507°  N 13.81511°  N 13.81397°  N 13.81330°  N 13.81543°  N 13.81397°  N 13.81511°  N 13.81543°  N 13.81330°  N 13.81330°  N 13.80930°  N 13.80756°  N 13.80763°  N 13.80763°  N 13.80834°  N 13.80756°  N 13.80930°  N 13.80864°˚  N 13.80930°  N 13.80756°  N 13.80763°  N 13.80864°  N 13.80930°  N 13.80780°  N 13.80930°  N 13.80864°  N 13.76080°  N 13.76180°  N 13.76080°  N 13.76180°  N 13.76080°  N 13.76180°  N 13.76180°  N 13.76180°  N 13.68977°  N 13.68977°  N 13.68977°  N 13.68786°  N 13.68977°  N 13.68977°  N 13.68977°  N 13.68977°  N 13.68977°  N 13.68786°  N 13.68952°  N 13.68952°  N 13.68952°  N 13.68977°  N 13.71879°  N 13.71840°  N 13.71840°  N 13.71840°  N 13.71840°  N 13.71840°  N 13.71942°  N 13.71942°  N 13.71942°  N 13.71899°  N 13.85263°  N 13.85327°  N 13.85326°  N 13.85331°  N 13.65106°  N 13.65106°  N 13.65122°  N 13.65137°  N 13.65137°  N 13.65110°  N 13.65110° | E 100.55470°  E 100.55404°  E 100.55470°  E 100.55297°  E 100.55559°  E 100.55404°  E 100.55258°  E 100.55404°  E 100.55559°  E 100.55258°  E 100.55559°  E 100.55258°  E 100.55258°  E 100.55258°  E 100.55559°  E 100.55487°  E 100.55297°  E 100.55487°  E 100.55297°  E 100.55608°  E 100.55202°  E 100.55276°  E 100.55326°  E 100.55202°  E 100.55326°  E 100.55202°  E 100.55326°  E 100.55326°  E 100.55202°  E 100.55376°  E 100.55202°  E 100.55383°  E 100.55276°  E 100.55370°  E 100.55383°  E 100.55202°  E 100.55370°  E 100.55276°  E 100.55276°  E 100.55008°  E 100.55186°  E 100.55264°  E 100.55264°  E 100.55086°  E 100.55186°  E 100.55025°  E 100.55055°  E 100.55008°  E 100.55186°  E 100.55264°  E 100.55055°  E 100.55025°  E 100.55379°  E 100.55008°  E 100.55055°  E 100.54031°  E 100.54051°  E 100.54031°  E 100.54051°  E 100.54031°  E 100.54051°  E 100.54051°  E 100.54051°  E 100.66517°  E 100.66517°  E 100.66517°  E 100.65989°  E 100.66517°  E 100.66517°  E 100.66517°  E 100.66517°  E 100.66517°  E 100.65989°  E 100.66489°  E 100.66489°  E 100.66489°  E 100.66517°  E 100.78114°  E 100.78114°  E 100.78114°  E 100.78114°  E 100.78114°  E 100.78114°  E 100.78056°  E 100.78056°  E 100.78056°  E 100.78087°  E 100.85923°  E 100.85883°  E 100.85873°  E 100.85927°  E 100.49097°  E 100.49097°  E 100.49033°  E 100.49037°  E 100.49037°  E 100.49060°  E 100.49060° | KU533691  KU533702  KU533711  KU533712  KU533656  KU533658  KU533670  KX382894  KU533655  KU533649  KU533673  KX382895  KU533643  KU533644  KU533713  KU533674  KU533675  KX382896  KU533695  KU533648  KU533676  KX382897  KX382898  KU533703  KX382899  KU533723  KX382900  KX382901  KU533704  KU533692  KU533693  KX382902  KU533694  KU533677  KX382903  KU533705  KU533678  KU533659  KU533679  KU533696  KU533680  KU533681  KU533682  KX382904  KU533683  KU533707  KU533660  KX382905  KU533684  KU533708  KU533706  KU533666  KU533697  KU533685  KX382906  KU533650  KU533714  KU533671  KU533651  KU533652  KU533709  KU533710  KU533641  KU533657  KU533686  KU533715  KU533716  KU533698  KU533717  KX382907  KU533699  KU533687  KU533661  KU533718  KU533700  KU533662  KU533720  KU533663  KU533653  KU533719  KU533688  KX382908  KU533672  KX382909  KU533654  KU533665  KU533645  KU533667  KU533689  KU533701  KU533664  KU533646  KU533647  KU533721  KU533669  KU533690  KU533642  KU533722 |
